# Chronic Cancer Pain: Opioids within Tumor Microenvironment Affect Neuroinflammation, Tumor and Pain Evolution

**DOI:** 10.3390/cancers14092253

**Published:** 2022-04-30

**Authors:** Angela Santoni, Matteo Santoni, Edoardo Arcuri

**Affiliations:** 1 Department of Molecular Medicine, Sapienza University of Rome, Laboratory Affiliated to Istituto Pasteur Italia-Fondazione Cenci Bolognetti, Viale Regina Elena 291, 00161 Rome, Italy; 2 IRCCS Neuromed, 86077 Pozzilli, Italy; 3 Medical Oncology Unit, Macerata General Hospital, Via Santa Lucia 2, 62100 Macerata, Italy; mattymo@alice.it; 4 IRCCS Regina Elena Cancer Institute, IFO, Via Elio Chianesi 53, 00128 Rome, Italy; edoardo.arcuri@fastwebnet.it; 5 Ars Medica Pain Clinic, Via Cesare Ferrero da Cambiano 29, 00191 Rome, Italy

**Keywords:** cancer pain, neuroinflammation, tumor microenvironment, opioid-induced hyperalgesia, immunotherapy

## Abstract

**Simple Summary:**

Pain is a worrisome symptom that 60–80% of patients with cancer experience chronically. In the last twenty years, immunological and pain research have shown that cancer pain is attributable to the neuroinflammatory response driven by the cellular and soluble components of the tumor microenvironment, with features similar to that induced in many other painful chronic non-cancer diseases. Neuroinflammation leads to central sensitization and neuroplastic remodeling of the central nervous system with alteration of pain sensitivity (hyperalgesia), responsiveness (behavior), and drive (centralization). Engagement of opioid receptors by both endogenous and exogenous opioids, namely, the cornerstone of pain therapy morphine, results in modulation of pain intensity and quality, in addition to cancer growth and progression. The effects of opioids on the evolution of pain, (relief or immune-mediated hyperalgesia) and cancer (promotion or inhibition), are dual and ambiguous. This ambiguity currently represents a major limitation of long-term opioid therapy, and encourages novel immunotherapeutic strategies.

**Abstract:**

Pain can be a devastating experience for cancer patients, resulting in decreased quality of life. In the last two decades, immunological and pain research have demonstrated that pain persistence is primarily caused by neuroinflammation leading to central sensitization with brain neuroplastic alterations and changes in pain responsiveness (hyperalgesia, and pain behavior). Cancer pain is markedly affected by the tumor microenvironment (TME), a complex ecosystem consisting of different cell types (cancer cells, endothelial and stromal cells, leukocytes, fibroblasts and neurons) that release soluble mediators triggering neuroinflammation. The TME cellular components express opioid receptors (i.e., MOR) that upon engagement by endogenous or exogenous opioids such as morphine, initiate signaling events leading to neuroinflammation. MOR engagement does not only affect pain features and quality, but also influences directly and/or indirectly tumor growth and metastasis. The opioid effects on chronic cancer pain are also clinically characterized by altered opioid responsiveness (tolerance and hyperalgesia), a hallmark of the problematic long-term treatment of non-cancer pain. The significant progress made in understanding the immune-mediated development of chronic pain suggests its exploitation for novel alternative immunotherapeutic approaches.

## 1. Chronic Cancer and Non-Cancer Pain as Disease in Itself

The WHO acknowledged the new vision of pain, and in 2015 a task force of the International Association for the Study of Pain (IASP) proposed a classification and codification of chronic pain within the International Classification of Diseases (ICD-11), both as disease and symptom [1,2]. This was the first time that pain was conceived as a disease in itself, and cancer pain was included in one of the seven categories of chronic painful conditions [3]. In addition, this new classification rationally framed all the chronic painful conditions: the proposed categories are currently considered exhaustive and mutually exclusive, and have relevant diagnostic value [4]. The recognition of chronic pain as disease does not represent only a simple taxonomic revolution, but, beyond the pathophysiological and clinical consequences, also implies a socio-economic impact that is finally proportionate to the enormous burden that chronic pain represents for 20% of the global population in the post-industrial civilization [5].

## 2. Is Cancer Pain “Different”?

Cancer is the major health-related cause of death worldwide [6], with 60–80% of cancer patients experiencing some degree of pain.

In the last twenty years, the immunological and pain research that had ignored each other for a long time, progressively interacted, so that immune cells were defined as circulating neurons, and the immune system as the “sixth sense” [7].

In the nineties, a collaboration started between the two authors of this review article, a pain therapist (EA) and an immunologist (AS), just when the nature of cancer pain, initially considered mainly dependent on “mechanic” stimulation, was shown to be attributable to the molecular mechanisms of neuroinflammation. Initial clinical (dynamic evolution of pain “quality”), biomolecular (expression of opioid receptors on cells other than neurons), and pharmacological (opioid-induced tolerance) features, raised then an intriguing question: is cancer pain somehow different? 

As we will discuss in this review article, the answer to this question has largely relied on the emerging insights on neuroinflammation and tumor microenvironment.

## 3. How Immunology Contributed to Modify the View of Chronic Pain

Chronic pain is defined by convention as pain that lasts more than three months after the initial insult (i.e., trauma, infection, inflammation), and is due to persistence of the causing event, lack of resolution, pain presence after seeming resolution, or even pain without a proven cause (*dolore sine materia*) [8]. Unlike acute pain, chronic pain has no clear physiological benefit as its perpetuation implies evolutive alterations of neuronal plasticity [9]. 

Neuroimmunology has provided seminal contributions demonstrating that the “*primum movens*” of transformation from acute to chronic pain, is neuroinflammation. This process that will be better described in the next section, involves a cross-talk between damage sensors (nociceptors of sensory neurons) and immune cells (neuro-immune network), with some of the receptors sensing danger signals common to both immune cells and sensory neurons [10]. The response to danger signals that is essential for returning to homeostasis in the short time of acute pain, when it persists, makes chronic pain “maladaptive”, by sensitizing the neuronal structures of input–output and lowering the pain threshold.

## 4. Neuroinflammation and Central Sensitization

In the last ten years, the number of publications on neuroinflammation and pain has increased by 10 times. Neuroinflammation is a localized form of inflammatory response triggered by different stressors and tissue injury (trauma, infection, chemotherapy, etc.), that occurs in the peripheral (PNS) and central nervous systems (CNS). The main features include: (a) increased vascular permeability of the brain–blood barrier; (b) immune cell recruitment and invasion in the CNS, previously considered an immunologically privileged site; (c) activation of glial cells (microglia, astrocytes and oligodendrocytes) and release of inflammatory mediators (i.e., cytokines, chemokines, growth factors, etc.) [11,12].

CNS infiltration by immune cells and activation of resident glial cells, in particular of microglia, are the tipping point of pain centralization, with remodeling of neuronal synapses in many brain areas that leads to “maladaptive” structural plasticity (changes in pain perception and behavior) [13]. Thus, neuroinflammation drives chronic pain via central sensitization. Glial cells are the hub of chronic pain, and their dysfunction in the spinal cord (microgliosis) observed in many chronic painful pathologies including cancer, is referred as “gliopathy” [14].

Notably, neuroinflammation has removed the vanishing border between cancer (malignant) and non-cancer (non-malignant) pain, and a new unifying vision of chronic pain has emerged: the real malignancy of pain is mainly due to the extent of neuroinflammation and neuroimmune dialogue that determines a disease within the disease. 

This more dynamic and holistic (psycho-social and emotional impact) view of pain went beyond the mechanistic and more static view, dividing pain in “*nociceptive”* (“due to noxius stimulation of non-neural tissue with a normal somatosensory nervous system”) and “*neuropathic*“ pain (“due to lesion or dysfunction of nervous system”) [15]. A third type of pain, “*nociplastic”* pain, that is characterized by “altered nociception and hypersensitivity with no clear evidence of tissue damage or disease or lesion of the somatosensory system”, as proposed by Kosek E., should overcome this dichotomy [16]. This third descriptor seems to be well-suited for the dynamic evolution of pain sensitivity.

## 5. The Complexity of Tumor Microenvironment 

The concept of tumor microenvironment dates back to more than a century ago, when the German physician Rudolf Virchow linked inflammation to cancer promotion, and described leukocyte infiltrates within tumors [17,18], and Paget formulated the theory of “seed and soil” [19]. 

It is now well established that the tumor microenvironment is a rather complex ecosystem in continuous evolution, with tumor cells functionally sculpting the surrounding stroma by releasing a wide array of soluble mediators, and/or mediating cell-cell interactions. In addition to tumor cells, the stroma also contains a large repertoire of distinct normal cells including inflammatory myeloid cells and lymphocytes, vascular cells, neurons, and fibroblasts that contribute to create the ‘‘tumor microenvironment’’ [20]. 

Immune cell infiltrates can exert both tumor-suppressive and tumor-promoting effects, and on the basis of their composition, activation, and/or functional states, tumors are referred as “hot” or “cold”. “Hot” tumors are characterized by T cell infiltration and accumulation of pro-inflammatory cytokines, and are associated with a favorable prognosis and better response rate to immune checkpoint therapy; conversely, “cold” tumors are rich in cells and molecules that inhibit antitumor responses and are associated with a worse prognosis [21].

Although tumor innervation was first reported in 1897 by Young H.H. [22], it is only recently that the nerve dependence in cancer has been described [23], and the PNS has been recognized as a crucial part of the tumor microenvironment. Development of nerves in the tumor microenvironment is required for cancer progression and dissemination, and this role, initially described in prostate cancer, was then reported in other tumor types including gastric, pancreatic, and non-melanoma skin cancer [24,25]. On the other hand, it is well established that cancer cells can surround and invade the epineurium and perineurium and reach the endoneurium, thus becoming intimately associated with Schwann cells and nerve axons. This process named *perineural invasion* is a common path of metastatic dissemination in many human cancers, and is associated with poor prognosis [26,27]. In this context, recent findings reported in mouse models, demonstrate that neural progenitor cells migrate from neurogenic niches within the brain to the tumor microenvironment, where they give rise to new nerve cells [28].

The mechanisms by which the neuroimmune axis affects cancer initiation and progression are still not fully elucidated. Release of neurotrophic growth factors by cancer cells or by tumor-infiltrating immune cells, has been suggested to promote nerve infiltration (axonogenesis), whereas release of neurotransmitters by nerve endings can stimulate cancer and stroma cell growth. 

## 6. Tumor Microenvironment as Scenario of Neuroinflammation and Chronic Pain 

How the cross-talk between nerves and cancer/immune/stromal cells contributes to the onset and persistence of cancer pain, is still elusive. Some of the molecular components involved in cancer pain and in particular in bone cancer pain, have been uncovered in in vivo preclinical studies. Schwei et al. [29] demonstrated that injection of mouse osteolytic sarcoma cells in the femur intramedullary space, resulted in confined tumor growth without soft tissue invasion, thus allowing the identification of neurochemical alterations occurring in the tumor microenvironment. Engagement of specific receptors on the nerve ending nociceptors by soluble inflammatory mediators, including ATP, protons, prostaglandins, endothelins, cytokines and neurotrophic growth factors, that are released by the cells forming the tumor microenvironment, can promote neuroinflammation, initiate signaling cascades affecting ion channel activity, transfer the signal to the spinal cord, and sensitize microglia and astrocytes. This induces lowering of the pain threshold with hyperalgesia and allodynia, resulting in long-term sensitization and pain centralization [30]. Implantation of different tumors in the bone tissue has generated distinct patterns of neuroinflammation and pain behaviors, suggesting that bone cancer pain involves multiple mechanisms, and is not only attributable to mechanic pressure exerted by the intramedullary growing tumor, as neurochemical reorganization of spinal cord, glial dysfunction and pain-related behaviors precede detectable bone destruction [31]. In addition, cancer progression is not always accompanied by a parallel pain exacerbation: in this regard, increased tumor vascularization and innervation, in addition to infiltration of nerve growth factor (NGF)-producing tumor-associated macrophages observed in a murine model of pancreatic cancer at earlier stage of disease, were not followed by worsening of painful behavior at a more advanced stage [32]. This inconsistency between disease progression and pain behavior might depend on the immune-mediated pain relief by endogenous opioids, a feedback mechanism to counteract the peripheral hyperalgesia in response to tissue injury, as described by Stein C. et al. [33].

Overall, the experimental models of bone cancer pain have been instrumental for better understanding the involvement of the neuroinflammatory response also in cancer pain. It is evident that bone cancer pain is a rather complex phenomenon that consists of inflammatory and neuropathic pain, and at the same time exhibits a unique “signature” in the CNS [34]. 

## 7. The Double-Edged Effects of Opioids and Their Receptors in the Tumor Microenvironment

In the last half century, exogenous opioids, namely, morphine and their derivates, have represented cornerstone treatments for cancer pain, although emerging immunological knowledge has clearly shown their ambiguity, in that these drugs do not only induce pain relief (antinociception), but can also exert opposite effects (pro-nociception, opioid-induced hyperalgesia, OIH) [35].

Opioid activity is mainly mediated by the typical seven transmembrane G protein coupled opioid receptors μ (MOR), δ (DOR), and κ (KOR) [36]. MOR is the principal receptor target for both endogenous (opiates) and exogenous opioids, and is mainly distributed in spinal nerve pathways, including the brainstem and the medial thalamus. MOR, however, is not only expressed by neuronal cells, but a large body of evidence indicates that it can also be detected on cancer, immune and endothelial cells present in the tumor microenvironment [37,38]. 

**Figure 1:**
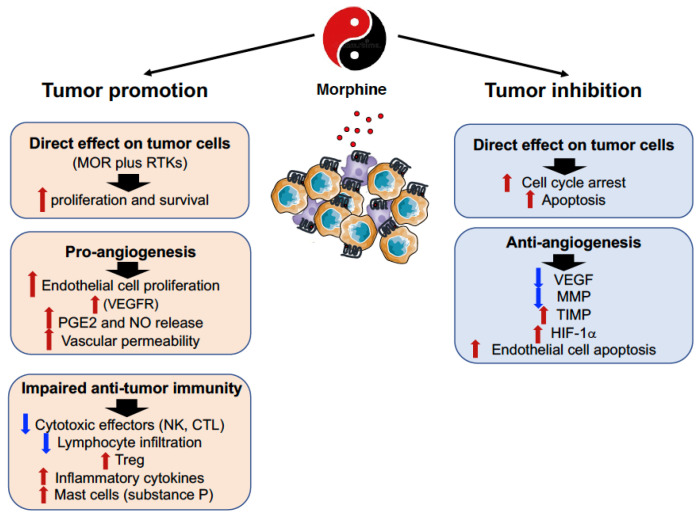
Double-edged effects of morphine in cancer. A large body of evidence indicates that morphine can either promote or inhibit tumor growth and metastasis formation, by direct effects on tumor cells, and by modulation of angiogenesis and anti-tumor immune responses. MOR, m opioid receptor; RTK, receptor tyrosine kinase; VEGFR, vascular endothelial growth factor receptor; PGE2, prostaglandin E2; NO, nitric oxide; NMDAR, N-methyl-D-aspartate receptor.

It is suggestive that the ambiguity of opioid effects on cancer pain is also evident on the modulation of tumor progression, and although we will discuss these issues separately, they are likely interdependent (Figure 1).

## 8. Opioids (Morphine) and MOR Interference with the Neoplastic Process

**Figure 2:**
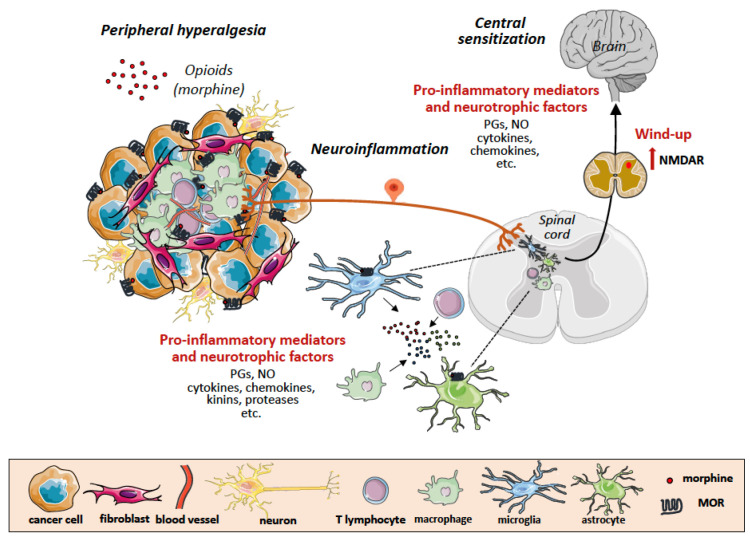
Tumor microenvironment as a scenario of neuroinflammation and central sensitization in chronic cancer pain. Binding of exogenous opioids to opioid receptors on nociceptors in the nerve endings, tumor cells and tumor-infiltrating leukocytes forming the tumor microenvironment, results in release of pro-inflammatory mediators and neutrophic factors that activate glial cells (microglia and astrocytes) in the dorsal root ganglion (DRG). Activated glial cells in turn also release pro-inflammatory mediators acting on neurons in the spinal cord and in the higher centers of brain, further propagating neuroinflammation and inducing central sensitization (wind-up and up-regulation of excitatory aminoacid (NMDA) receptors. MOR, m opioid receptor; PGE2, prostaglandin E2; NO, nitric oxide; NK, natural killer cell; CTL, cytotoxic T lymphocyte; Treg, regulatory T cell; VEGF, vascular endothelial growth factor; MMP, matrix metalloproteinase; TIMP, tissue inhibitor of metalloproteinase; HIF1a, hypoxia inducible factor 1 a.

The expression of MOR on tumor cells has been found in squamous cells of lung, breast, colon, liver, prostate, gastric and esophageal cancer both in preclinical models and in humans, and it has been associated with both promotion and inhibition of tumor growth and metastasis formation [39,40] (Figure 2). 

The pro-cancerous activity of MOR agonists, namely, morphine, can be attributable to direct effects on tumor cells in addition to modulation of angiogenesis and impairment of anti-tumor immune responses.

Morphine has been shown to promote tumor initiation in hepatocellular carcinoma (HCC) by regulating self-renewal of cancer stem cells and facilitating tumor cell proliferation [41]. Similarly, high expression of MOR on colorectal tumors was found to enhance cell proliferation, adhesion, migration, and tumorigenesis, while its inhibition delayed tumor development [42]. In vitro studies, however, reported that morphine largely fails to affect HT-29 colon cancer cell proliferation, while it causes increased secretion of the urokinase plasminogen activator, suggesting the ability of this opioid to promote the metastasizing ability of tumor cells [43]. On the other hand, morphine was also found to stimulate the proliferation of SH-SY5Y neuroblastoma cells, that only partially depended on MOR engagement. This effect was attributable to modulation of miRNAs, namely, *miR133b* and *miR128*, the target genes of which are involved in cytoskeletal reorganization, apoptosis, cell survival and proliferation [44]. 

With regard to the evidence that MOR can influence cancer progression and metastasis formation, human NSCLC cells treated with opioids, or MOR overexpression, exhibited an epithelial mesenchymal transition phenotype [45]. Moreover, in a transgenic mouse model that mimics the different steps of human breast cancer disease, morphine stimulated the progression of spontaneously developed tumors and shortened the survival of tumor-bearing mice. Morphine-induced mast cell activation contributed to cancer progression and also to refractory pain, by increasing the levels of inflammatory cytokines and substance P, a mediator known to stimulate angiogenesis and pain. Thus, mast cell activation by morphine may further exaggerate the pro-inflammatory, pro-nociceptive, and vasoactive tumor microenvironment [46].

In vivo, overexpression of MOR in human bronchoalveolar carcinoma cells increased primary tumor growth rates in nude mice by approximately 2.5-fold, and lung metastasis by approximately 20-fold as compared with vector control cells [47]. This tumor-promoting effect was associated with Akt and mTOR activation, and tumor cell proliferation, and extravasation.

Unlike these studies, exposure to morphine of the human cancer breast line MCF-7, resulted in inhibition of the expression of matrix metalloproteinases (MMP)-2 and -9 that are involved in the degradation of extracellular matrix, thus initiating the dissemination of invasive cancer cells [48]. Similarly, analysis of the circulating proteolytic profile in mice following morphine administration, demonstrated decreased MMP-9 and increased tissue inhibitor of metalloproteinase 1 (TIMP-1) and TIMP-3/4 with functional consequences on breast cancer cell migration and invasion [49].

A large body of evidence indicates that opioid-dependent tumor growth can also be secondary to the ability of these drugs to promote angiogenesis. The pro-angiogenic activity of morphine depends on the ability of this opioid to stimulate vascular endothelial growth factor (VEGF) receptor activation [50] and to initiate a signaling cascade leading to endothelial cell proliferation [51]. In addition, morphine results in the activation of cyclooxygenase 2 (COX-2) [52] and release of prostaglandin E2 that promotes angiogenesis and breast cancer progression [53]. Furthermore, opioid binding to MOR stimulates the generation of nitric oxide (NO) [54] that in turn activates COX with increased PGE2 production. 

Unlike these studies, morphine was also found to also inhibit tumor angiogenesis through the HIF-1α-p38-MAPK pathway [55], and to trigger endothelial cell apoptosis by increasing the generation of NO and of the reactive oxygen species, and decreasing mitochondrial membrane potential [56]. 

With regard to the direct effects of opioids on the immune system, they can be mediated via opioid and non-opioid toll-like receptors (see below). MOR expression was found on various immune cells, such as macrophages, neutrophils, dendritic cells (DC), NK cells, CD4^+^ and CD8^+^ T cells, and B cells [57]. Since 1979 when Wybran et al. reported [58] that morphine inhibited the rosetting of human peripheral blood T cells with sheep red blood cells, the activity of this opioid has been constantly demonstrated to be immunosuppressive, with impairment of both innate (neutrophil and macrophage phagocytosis and chemotaxis, natural killer cell cytotoxic activity, production of cytokines and chemokines), and adaptive (T and B cell proliferative responses to mitogens, cytokine production, modulation of regulatory T cells, antibody formation and secretion) immune responses [57,58,59,60]. Morphine can also inhibit several steps (sticking, rolling along blood vessel, etc.) of leukocyte migration and this activity might be responsible for altered immune cell infiltration into the tumor microenvironment [61].

Overall, the morphine-mediated suppressive effects on anti-tumor immune responses may play a central role in accelerating malignant tumor progression. 

Although the findings on the tumor-promoting activity of morphine are overwhelming, an area of increasing interest is emerging on the ability of this opioid to suppress tumor cell growth and progression. The anticancer activity of morphine has been shown both in in vitro cell cultures and in in vivo experiments. High doses of morphine were found to induce cell cycle arrest and apoptosis in different cell lines from *lung,* breast, hepatocellular and o*ral* squamous cell *carcinoma, neuroblastoma, and promyelocytic leukemia* [62,63,64,65,66]. Of interest, morphine-induced attenuation of breast cancer cell growth involved a rearrangement of the ErbB signaling network suggesting that morphine provides a promising strategy to enhance the sensitivity of breast cancer cells to ErbB-directed therapies [67]. Moreover, binding of morphine to the opioid growth factor receptor (OGFR), a negative regulator of normal and cancer cell proliferation [68], resulted in lung cancer growth suppression [69].

The ability of morphine to inhibit tumor growth and metastasis formation was also shown in numerous in vivo tumor models. Repeated administration of morphine suppressed tumor growth and metastasis in a mouse model of cancer pain produced by orthotopic inoculation of B16–BL6 melanoma cells into the hind paw [70]. Moreover, experimental lung metastasis upon intravenous injection of metastatic colon carcinoma cells were markedly reduced by subcutaneous morphine administration [71]. In nude mice, morphine significantly reduced the growth of human MCF-7 and MDA-MB231 breast adenocarcinoma cells, and this effect mainly relied on activation of p53 and induction of apoptosis [64]. In a similar xenograft model, inhibition of human gastric tumor growth was associated with morphine-induced decreased mRNA expression of NF-κB, and its target genes Bcl-2, cyclin D1, and VEGF [72]. 

In addition to MOR, it is now well established that morphine competitively binds to the accessory protein MD-2 associated with the innate immune receptor recognizing pathogen and damage molecules, TLR4 (Toll-like receptor 4); this receptor is not only expressed on immune cells, but is present also on glial cells and sensory neurons [10]. Morphine engagement of TLR4 initiates a signaling cascade leading to release of NO and production of inflammatory cytokines [73,74], that are responsible for opioid-induced neuroinflammation, central sensitization, and long-standing persistence of hyperalgesia after drug withdrawal. The implication of TLR4 in the morphine-mediated modulation of cancer initiation and progression has been unexplored so far [75], although this receptor is expressed on multiple cellular components of the tumor microenvironment. Of interest, morphine-induced upregulation of the inhibitory checkpoint protein PD-L1 (see below) on non-small cell lung cancer, was mediated via TLR4 and promoted tumor immune escape [76].

High MOR tumor expression has been associated with clinical severity and poor prognosis in patients with laryngeal carcinoma, [77], hepatocellular carcinoma [78], gastric cancer [79], and advanced prostate cancer [80], and several retrospective studies reported that patients who received general anesthesia with large amounts of opioids, show more cancer progression or recurrence than patients who received regional anesthesia or a lower amount of opioids [81,82,83]. Accordingly, administration of the MOR antagonist methylnaltrexone was found to be associated with longer survival time in cancer patients but not in healthy individuals [84]. However, the overall clinical impact to prevent or antagonize the opioid effects on cancer evolution appears a very problematic task that certainly warrants further investigation. In this regard, the American and European Societies of Regional Anesthesia excluded that there is sufficient evidence currently, to prefer regional anesthesia to reduce cancer recurrence [85]. 

The opposite effects of morphine on tumor development and progression may be partially explained by the experimental conditions used in the different studies (opioid doses and kinetics, tumor models, morphine plasma concentrations, etc). In addition, we have to keep in mind that in vitro and in vivo preclinical models do not take into account the genetic polymorphism and the epigenetic modifications of pain genes, in addition to opioid-induced phenomena such as tolerance and hyperalgesia, thus poorly reproducing the clinical reality. 

## 9. When and How MOR Became a Relevant Bridge between Pain and Immunological Research

The current integration between pain and immunological research would be hard to be understood without knowing some historical roots on the strong and often intriguing link among pain, cancer and opioids. 

In the eighties, the use of opioids for cancer pain was characterized by a tough polemic between supporters (the world of Cancer Palliative Care) and opponents (opiophobia). The opioids, morphine in particular, were considered, without ifs and buts, the “panacea” for the dramatic problem of cancer pain. At the same time, immunological research defined the endogenous counterpart of opioids, the opiates, as cytokines produced in response to danger signals and inflammatory stimuli [86]. At that time, the main underestimated opioid side effect was tolerance, namely, the requirement of continuous dosage escalation to counteract the progressive loss of therapeutic efficacy. The world of Palliative Care “instrumentally” argued that pain worsening was not attributable to drug tolerance, but rather to disease progression [87]. This “convenient” view poorly considered the well-established in vivo and in vitro evidence on the opioid receptor (MOR in particular) expression on cancer cells, that could evoke an unexpected and aberrant response on tumor progression. 

The awareness of these findings led us to explore in a xenograft tumor model whether morphine could modify cancer pain response, namely, tolerance, to the opioid. In accordance with the evidence on the expression of functional opioid receptors on tumor cells, we found that administration of morphine in tumor-bearing mice resulted in higher opioid concentration in the tumor tissue as compared with the normal counterpart, and exhibited a different pharmacodynamics in healthy vs tumor-bearing mice [88]. Furthermore, we also demonstrated that morphine binding to the opioid receptor(s) on tumor cells initiated a signaling cascade leading to activation of nitric oxide synthase and subsequent release of NO [89,90,91], a key mediator of neuroinflammation and central pain hypersensitivity [92]. These findings allowed us to suggest that the tumor microenvironment including all the cellular opioid receptor-expressing components (neuronal, immune, endothelial cells), acts as a functional trap, mimicking a peculiar kind of opioid tolerance [93]. In addition, some sporadic but increasing clinical evidence [94,95] indicated that the other side of opioid-induced tolerance was indeed OIH, the immune-mediated mechanisms of which would become disclosed many years later [35]. According to our idea, in the case of tumors with a prominent inflammatory component, chronic opioid exposure could lead to a peculiar form of breakthrough pain (unexplained spontaneous pain flares of rapid and sudden onset) [96,97]. Furthermore, the well-established NO involvement in amplifying pain hypersensitivity as a result of increased levels of excitatory amino acids (N-methyl-D-aspartate, NMDA) in the CNS (wind-up), and the ability of methadone also to stimulate NO generation, prompted us to wonder whether structurally diverse clinically employed opioid analgesics could exert the same activity. We found that the ability of different opioids to trigger NO release in MOR-expressing glioblastoma cells, paralleled the extent of tolerogenic and hyperalgesic effects induced by each opioid, being morphine at the top of the list and methadone at the last place (and, therefore, considered a reference drug for addiction maintaining therapy) [98]. Only a few years later, morphine-induced hyperalgesia was also linked to its ability to bind to TLR-4 [73,74] on the spinal cord microglia, that initially determines the extent of neuroinflammatory response and then the persistence of both cancer and non-cancer pain, depending on injury intensity and quality. 

In our opinion, the above-mentioned findings together with numerous clinical observations strongly support the idea that overlapping mechanisms underlie cancer and non-cancer pain, in addition to OIH [15].

## 10. Cancer Immunotherapy and Pain

Over the past decade, treatments promoting anti-tumor immune responses have revolutionized cancer therapy [99]. Antibodies targeting the inhibitory checkpoint proteins CTLA-4, PD-1, or the PD-1 ligand PDL-1, have been approved for treatment of a variety of cancers, including melanoma, non-small-cell lung cancer, head and neck cancer, bladder cancer, renal cell carcinoma, hepatocellular carcinoma, and several other tumor types, and have resulted in marked and durable responses [100]. 

However, only little evidence on the effects of these novel immunotherapeutic approaches on pain are available, and they mainly stem from preclinical studies. In mouse models of neuropathic and cancer pain, the checkpoint pathway ligand PD-L1 was found to inhibit pain and allodynia by suppressing basal pain sensitivity upon engagement and activation of its cognate receptor PD-1 on peripheral sensory neurons [101]. Stimulation of PD-L1-PD-1 counter-pair signaling resulted in activation of the tyrosine-phosphatase SHP-1, leading to reduced pain-sensing neuron excitability and downstream modulation of sodium and potassium channels. Similarly, local injection of PDL-1 was reported to activate SHP-1 that co-localizes with PD-1 and with the transient receptor potential vanilloid 1 (TRPV1) in dorsal root ganglion (DRG) neurons; this results in downregulation of receptor channel activity, and inhibition of bone cancer pain development in mice inoculated with Lewis lung carcinoma cells [102]. The PD-1-PD-1L-produced analgesic effect on murine bone cancer was attributable to the ability of the immune checkpoint pathway to inhibit RANK-L-induced osteoclastogenesis [103]. Moreover, Wang Z. et al. reported that anti-PD-1 treatment impairs opioid-induced antinociception in rodents and nonhuman primates, as PD-1 is co-expressed with MOR in sensory and DRG neurons and is required for MOR signaling. PD-1 blockade suppressed calcium currents, excitatory synaptic transmission, and induction of outward currents in spinal cord neurons in addition to enhanced opioid-induced hyperalgesia and tolerance, and potentiated opioid-induced microgliosis and long-term potentiation in the spinal cord. In addition, intrathecal infusion of the anti-PD-1 antibody nivolumab, inhibited intrathecal morphine-induced antinociception in nonhuman primates [104]. 

Collectively, these findings suggest that anti-PD-1 immunotherapy interferes with opioid analgesia in patients with cancer by disrupting the PD-1–MOR interaction.

Although preclinical studies highlight that the immune checkpoint blockade therapy may produce long-term benefits in cancer pain, only anecdotal findings are present in the clinical setting, and large controlled clinical trials specifically addressing this issue, are lacking so far [105]. 

Indeed, mononeuritis multiplex and back pain as a complication of combined therapy with the anti-CTL-4 (ipilimumab) and anti-PD-1 (nivolumab) monoclonal antibodies, was were described in a melanoma patient [106]. Similarly, neuropathic pain was associated with chronic inflammatory demyelinating polyradiculoneuropathy secondary to immune checkpoint inhibitors in two melanoma patients [107]. Moreover, in an observational cohort study enrolling 162 melanoma or non-small lung cancer patients treated with PDL-1 inhibitors, chest and abdominal pain emerged among the most common patient-reported clinically relevant symptoms [108]. Of interest, a recent case report also showed that PD-1 immunotherapy elicited severe itch (pruritus) [109], a symptom that shares the pain neural circuits [110], in a patient with a 7-month history of lung adenocarcinoma; remarkably, treatment with naloxone resulted in substantial relief within 1 h, suggesting a correlation between PD-1 and MOR in humans. 

## 11. Immunotherapy of Pain

**Figure 3:**
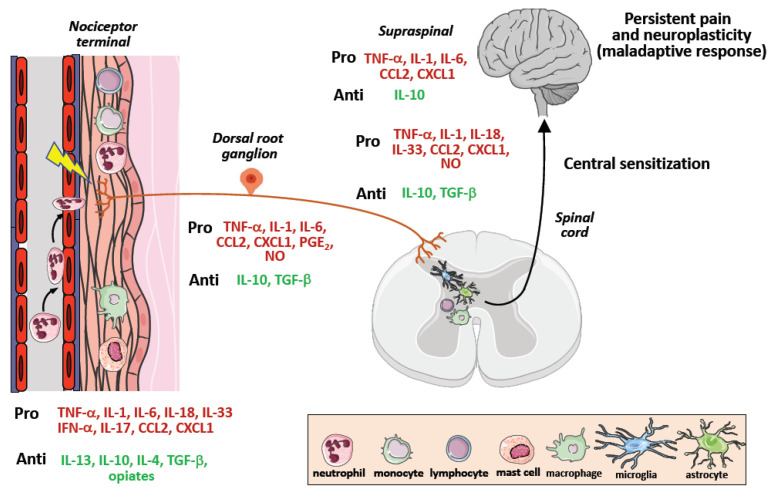
Pro-inflammatory (pro-nociceptive) and anti-inflammatory (antinociceptive) mediators are released during chronic pain development. Upon tissue damage or infection, cytokines are released locally by tissue resident or blood-recruited immune cells. The peripheral terminals of nociceptors, dorsal root ganglia and spinal cord express several receptors for these mediators, the signaling of which modulates nociceptive activity. Cytokines are displayed as pronociceptive (red) or antinociceptive (green).

Because of the numerous evidence on the critical role of immune cells as key orchestrators of pain due to their ability to infiltrate neuronal tissues and release molecular mediators sensitizing nociceptor neurons [111], a number of therapeutic strategies based on the modulation of neuroinflammatory and neuroimmune responses have been developed, although they mainly regard preclinical pain models of non-cancer neuropathic chronic pain [112]. These approaches consist of targeting neuroinflammation, by neutralization of proinflammatory mediators such as TNF-α, IL-6, IL-1, IL-18, IL-33, and CCL2, or administration of anti-inflammatory mediators including IL-10, IL-4, IL-13, and TGF-β [113,114], and resolvins [115] (Figure 3).

In addition, cellular immunotherapeutic approaches exploiting the ability of macrophages and T cells to infiltrate spinal cord [111], have also been taken into consideration. Of interest, the study by Pannell M et al. showed in a model of neuropathic pain that perineural transplantation of IL-4-induced anti-inflammatory M2 macrophages secreting higher levels of endogenous opioids at the damaged nerves, reduced neuropathy-induced tactile hypersensitivity in vivo, thus mimicking immune-mediated peripheral analgesia [116,117]. 

In terms of T cells, cisplatin educated CD8 T cells were found to prevent and resolve chemotherapy-induced peripheral neuropathy in mice [118]. In addition, reduced cancer pain severity was observed in advanced cancer patients following adoptive immunotherapy with infusion of autologous T cells [119]. 

## 12. Conclusions

The third millennium has reassembled in a unified view the diverse pathogenetic mechanisms of chronic pain that persists beyond the evoking cause. The synthetically crude definition of cancer as “the wound that does not heal”, could also be adopted to its main symptom, chronic pain, of which the “dignity” of disease in itself has been finally recognized. The involvement of neuroinflammation in pain initiation, of spinal cord infiltrating immune cells in pain sensitization, and the neuroplastic “maladaptive alterations” occurring in the brain, are common events in cancer and non-cancer pain [15]. Therefore, chronic cancer pain does not seem to be so unique. However, the composition of the tumor microenvironment, a complex ecosystem where different cell types and soluble mediators vary during the neoplastic process, suggests that distinct neuroinflammatory responses for each tumor type and stage, generate different pain states. Today we also know that the different cellular components forming the tumor microenvironment express opioid receptors (MOR in particular) that once engaged by endogenous (opiates) and exogenous opioids, may mediate unpredictable effects on pain and tumor evolution. Indeed, long-term opioid (morphine in particular) therapy results in paradoxical effects (pain worsening) that create a differential diagnostic dilemma between tolerance, OIH and disease progression. In case of OIH, opioid escalation represents the devastating choice of curing the effect (pain) with the cause (opioids), thus transforming the “therapy of pain” into “pain from therapy”. 

Perhaps the dramatic prevalence of cancer pain has fostered the underestimation of the exogenous opioid capacity to hijack the neuroimmune network controlled by the endogenous opioid system (bona fide cytokines) [120]. Such underestimation has likely been the basis of the phenomenon of opioid epidemics in the United States and Canada [121]. 

The opioid effects on tumor evolution are also ambiguous. These studies have been mainly carry out in preclinical models and show a number of limitations: 1. The animal tumor models poorly reproduce the complexity of the human disease; 2. The contradictory results on tumor growth and metastasis dissemination are likely to be a reflection of the different experimental models and methodologies employed; 3. The lack of correlation between the opioid dosage employed in in vitro assays [122] and that administered in in vivo experimental models and in the clinical setting; 4. The failure to correlate the tolerogenic effects observed in the animal models with the pharmacological and psychological (emotional) effects occurring in humans [83]. 

Thus, based on the enormous amount of experimental evidence on the dual effects of opioids on both pain and cancer, the clinical outcome of long-term opioid pain therapy would be difficult to predict also for an expert in the field: pain relief and its benefits, or rather, disease worsening and reduced life expectancy. 

Currently, opioid ambiguity represents an important emerging limitation [123,124], but it could also be an incentive to design novel opioid and non opioid drugs, and to promote an interdisciplinary science fact between Immunology and Algology, that we defined—between the serious and the ironic—as ImmunoAlgology [125].

## References

[1] Treede R.D., Rief W., Barke A., Aziz Q., Bennett M.I., Benoliel R., Cohen M., Evers S., Finnerup N.B., First M.B. (2019). Chronic pain as a symptom or a disease: The IASP Classification of Chronic Pain for the International Classification of Diseases (ICD-11). Pain.

[2] Nicholas M., Vlaeyen J.W.S., Rief W., Barke A., Aziz Q., Benoliel R., Cohen M., Evers S., Giamberardino M.A., Goebel A. (2019). The IASP classification of chronic pain for ICD-11: Chronic primary pain. Pain.

[3] Bennett M.I., Kaasa S., Barkee A., Korwisie B., Riefe W., Treede R., The IASP Taskforce for the Classification of Chronic Pain (2019). The IASP classification of chronic pain for ICD-11: Chronic cancer-related pain. Pain.

[4] Barke A., Korwisi B., Hans-Raimund C., Fors E.A., Geber C., Schug S.A., Stubhaug A., Ushida T., Wetterling T., Rief W. (2018). Pilot field testing of the chronic pain classification for ICD-11: The results of ecological coding. BMC Public Health.

[5] Breivik H., Cherny N., Collett B., de Conno F., Filbet M., Foubert A.J., Cohen R., Dow L. (2009). Cancer-related pain: A pan-European survey of prevalence, treatment, and patient attitudes. Ann. Oncol..

[6] Ferlay J., Colombet M., Soerjomataram I., Parkin D.M., Piñeros M., Znaor A., Bray F. (2021). Cancer statistics for the year 2020: An overview. Int. J. Cancer.

[7] Blalock J.E., Smith E.M. (2007). Conceptual development of the immune system as a sixth sense. Brain Behav. Immun..

[8] Basbaum A.I., Bautista D.M., Scherrer G., Julius D. (2009). Cellular and molecular mechanisms of pain. Cell.

[9] Woolf C.J. (2011). Central sensitization: Implications for the diagnosis and treatment of pain. Pain.

[10] Donnelly C.R., Chen O., Ji R.R. (2020). How do sensory neurons sense danger signals?. Trends Neurosci..

[11] Grace P.M., Hutchinson M.R., Maier S.F., Watkins L.R. (2014). Pathological pain and the neuroimmune interface. Nat. Rev. Immunol..

[12] Ji R.R., Nackley A., Huh Y., Terrando N., Maixner W. (2018). Neuroinflammation and central sensitization in chronic and widespread pain. Anesthesiology.

[13] Kuner R., Flor H. (2016). Structural plasticity and reorganisation in chronic pain. Nat. Rev. Neurosci..

[14] Ji R.R., Berta T., Nedergaard M. (2013). Glia and pain: Is chronic pain a gliopathy?. Pain.

[15] Santoni A., Mercadante S., Arcuri E. (2021). Chronic cancer and non-cancer pain and opioid-induced hyperalgesia share common mechanisms: Neuroinflammation and central sensitization. Minerva Anestesiol..

[16] Kosek E., Cohen M., Baron R., Gebhart G.F., Mico J.A., Rice A.S., Rief W., Sluka A.K. (2016). Do we need a third mechanistic descriptor for chronic pain states?. Pain.

[17] Virchow R.A. (1858). Die Cellular Pathologie in Ihrer Begründung auf Physiologische und Pathologische Gewebelehre.

[18] Balkwill F., Mantovani A. (2001). Inflammation and cancer: Back to Virchow?. Lancet.

[19] Paget S. (1889). The distribution of secondary growths in cancer of the breast. Lancet.

[20] Maman S., Witz I.P. (2018). A history of exploring cancer in context. Nat. Rev. Cancer.

[21] Fridman W.H., Zitvogel L., Sautès-Fridman C., Kroemer G. (2017). The immune contexture in cancer prognosis and treatment. Nat. Rev. Clin. Oncol..

[22] Young H.H. (1897). On the presence of nerves in tumors and of other structures in them as revealed by a modification of Ehrlich’s method of “vital staining” with methylene blue. J. Exp. Med..

[23] Boilly B., Faulkner S., Jobling P., Hondermarck H. (2017). Nerve dependence: From regeneration to cancer. Cancer Cell.

[24] Selvaraj D., Kuner R. (2015). Molecular players of tumor-nerve interactions. Pain.

[25] Jobling P., Pundavela J., Oliveira M.R.S., Roselli S., Walker M.M., Hondermarck H. (2015). Nerve–Cancer Cell Cross-talk: A Novel Promoter of Tumor Progression. Cancer Res..

[26] Amit M., Na’ara S., Gil Z. (2016). Mechanisms of cancer dissemination along nerves. Nat. Rev. Cancer.

[27] Marchesi F., Piemonti L., Mantovani A., Allavena P. (2010). Molecular mechanisms of perineural invasion, a forgotten pathway of dissemination and metastasis. Cytokine Growth Factor Rev..

[28] Mauffrey P., Tchitchek N., Barroca V., Bemelmans A.P., Firlej V., Allory Y., Roméo P.H., Magnon C. (2019). Progenitors from the central nervous system drive neurogenesis in cancer. Nature.

[29] Schwei M.J., Honore P., Rogers S.D., Salak-Johnson J.L., Finke M.P., Ramnaraine M.L., Clohisy D.R., Mantyh P.W. (1999). Neurochemical and cellular reorganization of the spinal cord in a murine model of bone cancer pain. J. Neurosci..

[30] Mantyh P.W., Clohisy D.R., Koltzenburg M., Hunt S.P. (2002). Molecular mechanisms of cancer pain. Nat. Rev. Cancer.

[31] Sabino M.A., Luger N.M., Mach D.B., Rogers S.D., Schwei M.J., Mantyh P.W. (2003). Different tumors in bone each give rise to a distinct pattern of skeletal destruction, bone cancer-related pain behaviors and neurochemical changes in the central nervous system. Int. J. Cancer.

[32] Mantyh P.W. (2006). Cancer pain and its impact on diagnosis, survival and quality of life. Nat. Rev. Neurosci..

[33] Stein C., Schäfer M., Machelska H. (2003). Attacking pain at its source: New perspectives on opioids. Nat. Med..

[34] Luger N.M., Sabino M.A., Schwei M.J., Mach D.B., Pomonis J.D., Keyser C.P., Rathbun M., Clohisy D.R., Honore P., Yaksh T.L. (2002). Efficacy of systemic morphine suggests a fundamental difference in the mechanisms that generate bone cancer vs inflammatory pain. Pain.

[35] Mercadante S., Arcuri E., Santoni A. (2019). Opioid-induced tolerance and hyperalgesia. CNS Drugs.

[36] Stein C. (2016). Opioid Receptors. Annu. Rev. Med..

[37] Scroope C.A., Singleton Z., Hollmann M.W., Parat M.O. (2021). Opioid receptor-mediated and non-opioid receptor-mediated roles of opioids in tumour growth and metastasis. Front. Oncol..

[38] Amaram-Davila J., Davis M., Reddy A. (2020). Opioids and cancer mortality. Curr. Treat. Options Oncol..

[39] Zhang X.Y., Liang X.Y., Yan Y., Dai Z., Chu H.C. (2018). Morphine: Double-faced roles in the regulation of tumor development. Clin. Transl. Oncol..

[40] Tuerxun H., Cui J. (2019). The dual effect of morphine on tumor development. Clin. Transl. Oncol..

[41] Li Y., Li G., Tao T., Kang X., Liu C., Zhang X., Wang C., Li C., Guo X. (2019). The μ-opioid receptor (MOR) promotes tumor initiation in hepatocellular carcinoma. Cancer Lett..

[42] Lu H., Zhang H., Weng M.L., Zhang J., Jiang N., Cata J.P., Ma D., Chen W.K., Miao C.H. (2021). Morphine promotes tumorigenesis and cetuximab resistance via EGFR signaling activation in human colorectal cancer. J. Cell. Physiol..

[43] Tagirasa R., Yoo E. (2022). Role of Serine Proteases at the Tumor-Stroma Interface. Front. Immunol..

[44] Gonzalez-Nunez V., Noriega-Prieto J.A., Rodriguez R.E. (2014). Morphine modulates cell proliferation through mir133b & mir128 in the neuroblastoma SH-SY5Y cell line. Biochim. Biophys. Acta.

[45] Lennon F.E., Mirzapoiazova T., Mambetsariev B., Poroyko V.A., Salgia R., Moss J., Singleton P.A. (2014). The Mu Opioid Receptor Promotes Opioid and Growth Factor-Induced Proliferation, Migration and Epithelial Mesenchymal Transition (EMT) in Human Lung Cancer. PLoS ONE.

[46] Nguyen J., Luk K., Vang D., Soto W., Vincent L., Robiner S., Saavedra R., Li Y., Gupta P., Gupta K. (2014). Morphine stimulates cancer progression and mast cell activation and impairs survival in transgenic mice with breast cancer. Br. J. Anaesth..

[47] Lennon F.E., Mirzapoiazova T., Mambetsariev B., Salgia R., Moss J., Singleton P.A. (2012). Overexpression of the m-Opioid Receptor in Human Non-Small Cell Lung Cancer Promotes Akt and mTOR Activation, Tumor Growth, and Metastasis. Anesthesiology.

[48] Gach K., Szemraj J. (2011). The influence of opioids on matrix metalloproteinase-2 and -9 secretion and mRNA levels in MCF-7 breast cancer cell line. Mol. Biol. Rep..

[49] Xie N., Khabbazi S., Nassar Z.D., Gregory K., Vithanage T., Anand-Apte B., Cabot P.J., Sturgess D., Shaw P.N., and Parat M. (2017). Morphine alters the circulating proteolytic profile in mice: Functional consequences on cellular migration and invasion. FASEB J..

[50] Singleton P.A., Lingen M.W., Fekete M.J., Garcia J.G., Moss J. (2006). Methylnaltrexone inhibits opiate and VEGF-induced angiogenesis: Role of receptor transactivation. Microvasc. Res..

[51] Gupta K., Kshirsagar S., Chang L., Schwartz R., Law P.Y., Yee D., Hebbel R.P. (2002). Morphine stimulates angiogenesis by activating proangiogenic and survival-promoting signaling and promotes breast tumor growth. Cancer Res..

[52] Farooqui M., Li Y., Rogers T., Poonawala T., Griffin R.J., Song C.W., Gupta K. (2007). COX-2 inhibitor celecoxib prevents chronic morphine-induced promotion of angiogenesis, tumour growth, metastasis and mortality, without compromising analgesia. Br. J. Cancer.

[53] Chang S., Liu C.H., Conway R., Han D.K., Nithipatikom K., Trifan O.C., Lane T.F., Hla T. (2004). Role of prostaglandin E2-dependent angiogenic switch in cyclooxygenase 2-induced breast cancer progression. Proc. Natl. Acad. Sci. USA.

[54] Rasmussen M., Zhu W., Tønnesen J., Cadet P., Tønnesen E., Stefano G.B. (2002). Effects of morphine on tumour growth. Neuroendocrinol. Lett..

[55] Koodie L., Ramakrishnan S., Roy S. (2010). Morphine suppresses tumor angiogenesis through a HIF-1alpha/p38MAPK pathway. Am. J. Pathol..

[56] Hsiaoa P., Changa M., Cheng W., Chen C., Lin H., Hsieh C., Sun W. (2009). Morphine induces apoptosis of human endothelial cells through nitric oxide and reactive oxygen species pathways. Toxicology.

[57] Eisenstein T.K. (2019). The Role of Opioid Receptors in Immune System Function. Front. Immunol..

[58] Wybran J., Appelboom T., Famaey J.P., Govaerts A. (1979). Suggestive evidence for receptors for morphine and methionine-enkephalin on normal human blood T lymphocytes. J. Immunol..

[59] Plein L.M., Rittner H.L. (2018). Opioids and the immune system—Friend or foe. Br. J. Pharmacol..

[60] Gong L., Dong C., Ouyang W., Quin Q. (2013). Regulatory T cells: A possible promising approach to cancer recurrence induced by morphine. Med. Hypotheses.

[61] Koodie L., Yuan H., Pumper J.A., Yu H., Charboneau R., Ramkrishnan S. (2014). Morphine inhibits migration of tumor-infiltrating leukocytes and suppresses angiogenesis associated with tumor growth in mice. Am. J. Pathol..

[62] Kawase M., Sakagami H., Furuya K., Kikuchi H., Nishikawa H., Motohashi N., Morimoto Y., Varga A., Molnar J. (2002). Cell death-inducing activity of opiates in human oral tumor cell lines. Anticancer Res..

[63] Sueoka E., Sueoka N., Kai Y., Okabe S., Suganuma M., Kanematsu K., Yamamoto T., Fujiki H. (1998). Anticancer activity of morphine and its synthetic derivative, KT-90, mediated through apoptosis and Inhibition of NF-kB activation. Biochem. Biophys. Res. Commun..

[64] Chen Y., Qin Y., Li L., Chen J., Zhang X., Xie Y. (2017). Morphine can inhibit the growth of breast cancer MCF-7 cells by arresting the cell cycle and inducing apoptosis. Biol. Pharm. Bull..

[65] Gach K., Wyrębska A., Fichna J., Janecka A. (2011). The role of morphine in regulation of cancer cell growth. Arch. Pharmacol..

[66] Weingaertner I.R., Koutnik S., Ammer H. (2013). Chronic morphine treatment attenuates cell growth of human BT474 breast cancer cells by rearrangement of the ErbB signalling network. PLoS ONE.

[67] Zagon I.S., Verderameb M.F., McLaughlin P.J. (2002). The biology of the opioid growth factor receptor (OGFr). Brain Res. Rev..

[68] Kim J.Y., Ahn H.J., Kim J.K., Kim J., Lee S.H., Chae H.B. (2016). Morphine suppresses lung cancer cell proliferation through the interaction with opioid growth factor receptor: An in vitro and human lung tissue study. Anesth. Analg..

[69] Sasamura T., Nakamura S., Iida Y., Fujii H., Murata J., Saiki I., Nojima H., Kuraishi Y. (2002). Morphine analgesia suppresses tumor growth and metastasis in a mouse model of cancer pain produced by orthotopic tumor inoculation. Eur. J. Pharmacol..

[70] Harimaya Y., Koizumi K., Andoh T., Nojima H., Kuraishi Y., Saiki I. (2002). Potential ability of morphine to inhibit the adhesion, invasion and metastasis of metastatic colon 26-L5 carcinoma cells. Cancer Lett..

[71] Tegeder I., Grosch S., Schmidtko A., Haussler A., Schmidt H., Niederberger E., Scholich K., Geisslinger G. (2003). G protein-independent G1 cell cycle block and apoptosis with morphine in adenocarcinoma cells: Involvement of p53 phosphorylation. Cancer Res..

[72] Li C., Li L., Qin Y., Jiang Y., Wei Y., Chen J., Yubo X. (2020). Exogenous morphine inhibits the growth of human gastric tumor in vivo. Ann. Transl. Med..

[73] Wang X., Loram L.C., Ramos K., de Jesus A.J., Thomas J., Cheng K., Reddy A., Somogyi A.A., Hutchinson M.R., Watkins L.R. (2012). Morphine activates neuroinflammation in a manner parallel to endotoxin. Proc. Natl. Acad. Sci. USA.

[74] Watkins L.R., Hutchinson M.R., Rice K.C., Maier S.F. (2009). The “toll” of opioid-induced glial activation: Improving the clinical efficacy of opioids by targeting glia. Trends Pharmacol. Sci..

[75] Gabr M.M., Saeed I., Miles J.A., Ross B.P., Shaw P.N., Hollmann M.W., Parat M.O. (2021). Interaction of opioids with TLR4-mechanisms and ramifications. Cancers.

[76] Wang K., Wang J., Liu T., Yu W., Dong N., Zhang C., Xia W., Wei F., Yang L., Ren X. (2021). Morphine-3-glucuronide upregulates PD-L1 expression *via* TLR4 and promotes the immune escape of non-small cell lung cancer. Cancer Biol. Med..

[77] Zhang H., Sun M., Zhou D., Gorur A., Sun Z., Zeng W., Cata J.P., Chen W., Miao C. (2020). Increased mu-opioid receptor expression is associated with reduced disease-free and overall survival in laryngeal squamous cell carcinoma. Br. J. Anaesth..

[78] Chen D.T., Pan J.H., Chen Y.H., Xing W., Yan Y., Yuan Y.F., Zeng W.A. (2019). The mu-opioid receptor is a molecular marker for poor prognosis in hepatocellular carcinoma and represents a potential therapeutic target. Br. J. Anaesth..

[79] Yao Y.S., Yao R.Y., Zhuang L.K., Qi W.W., Lv J., Zhou F., Qiu W.S., Yue L. (2015). MOR1 expression in gastric cancer: A biomarker associated with poor outcome. Clin. Transl. Sci..

[80] Zylla D., Gourley B.L., Vang D., Jackson S., Boatman S., Lindgren B., Kuskowski M.A., Le C., Gupta K., Gupta P. (2013). Opioid requirement, opioid receptor expression, and clinical outcomes in patients with advanced prostate cancer. Cancer.

[81] Cata J.P. (2018). Outcomes of regional anesthesia in cancer patients. Curr. Opin. Anaesthesiol..

[82] Forget P., Aguirre J.A., Bencic I., Borgeat A., Cama A., Condron C., Eintrei C., Eroles P., Gupta A., Hales T.G. (2019). How anesthetic, analgesic and other non-surgical techniques during cancer surgery might affect postoperative oncologic outcomes: A summary of current state of evidence. Cancers.

[83] Novy D.M., Nelson D.V., Koyyalagunta D., Cata J.P., Gupta P., Gupta K. (2020). Pain, opioid therapy, and survival: A needed discussion. Pain.

[84] Janku F., Johnson L.K., Karp D.D., Atkins J.T., Singleton P.A., Moss J. (2016). Treatment with methylnaltrexone is associated with increased survival in patients with advanced cancer. Ann Oncol..

[85] Missair A., Cata J.P., Votta-Velis G., Johnson M., Borgeat A., Tiouririne M., Gottumukkala V., Buggy D., Vallejo R., Marrero E.B. (2019). Impact of perioperative pain management on cancer recurrence: An ASRA/ESRA special article. Reg. Anesth. Pain Med..

[86] Peterson P.K., Molitor T.W., Chao C.C. (1998). The opioid–cytokine connection. J. Neuroimmunol..

[87] Collin E., Poulain P., Gauvain-Piquard A., Petit G., Pichard-Leandri E. (1993). Is disease progression the major factor in morphine “tolerance” in cancer pain treatment?. Pain.

[88] Arcuri E., Ginobbi P., Tirelli W., Froldi R., Citro G., Santoni A. (2002). Preliminary in vivo experimental evidence on intratumoral morphine uptake. Possible clinical implications in cancer pain and opioid responsiveness. J. Pain Symptom Manag..

[89] Pasternak G.W., Kolesnikov Y.A., Babey A.M. (1995). Perspectives on the N-methyl-D-aspartate/nitric oxide cascade and opioid tolerance. Neuropsychopharmacology.

[90] Kolesnikov Y.A., Pick C.G., Ciszewska G., Pasternak G.W. (1993). Blockade of tolerance to morphine but not to kappa opioids by a nitric oxide synthase inhibitor. Proc. Natl. Acad. Sci. USA.

[91] Fimiani C., Arcuri E., Santoni A., Rialas C.M., Bilfinger T.V., Peter D., Salzet B., Stefano G.B. (1999). Mu3 opiate receptor expression in lung and lung carcinoma: Ligand binding and coupling to nitric oxide release. Cancer Lett..

[92] DeLeo J.A., Yezierski R.P. (2001). The role of neuroinflammation and neuroimmune activation in persistent pain. Pain.

[93] Arcuri E. (1998). Can tumors act as opioid traps, mimicking opioid tolerance?. J. Pain Symptom Manag..

[94] Sjøgren P., Jensen N.H., Jensen T.S. (1994). Disappearance of morphine-induced hyperalgesia after discontinuing or substituting morphine with other opioid agonists. Pain.

[95] Mercadante S., Ferrera P., Villari P., Arcuri E. (2003). Hyperalgesia: An emerging iatrogenic syndrome. J. Pain Symptom Manag..

[96] Mercadante S., Arcuri E. (1998). Breakthrough pain in cancer patients: Pathophysiology and treatment. Cancer Treat. Rev..

[97] Arcuri E., Ginobbi P., Tirelli W., Ayers G., Milana R. (2012). Breakthrough pain: A single mask for varying painful situations: Therapeutic reflexes in cancer pain. Transl. Med..

[98] Mastronicola D., Arcuri E., Arese M., Bacchi A., Mercadante S., Cardelli P., Citro G., Sarti P. (2004). Morphine but not fentanyl and methadone affects mitochondrial membrane potential by inducing nitric oxide release in glioma cells. Cell. Mol. Life Sci..

[99] Ribas A., Wolchok J.D. (2018). Cancer immunotherapy using checkpoint blockade. Science.

[100] Esfahani K., Roudaia L., Buhlaiga N., Del Rincon S.V., Papneja N., Miller W.H. (2020). A review of cancer immunotherapy: From the past, to the present, to the future. Curr. Oncol..

[101] Chen G., Kim Y.H., Li H., Luo H., Liu D.L., Zhang Z.J., Lay M., Chang W., Zhang Y.Q., Ji R.R. (2017). PD-L1 inhibits acute and chronic pain by suppressing nociceptive neuron activity via PD-1. Nat. Neurosci..

[102] Liu B.L., Cao Q.L., Zhao X., Liu H.Z., Zhang Y.Q. (2020). Inhibition of TRPV1 by SHP-1 in nociceptive primary sensory neurons is critical in PD-L1 analgesia. JCI Insight.

[103] Wang K., Gu Y., Liao Y., Bang S., Donnelly C.R., Chen O., Tao X., Mirando A.J., Hilton M.J., Ji R.R. (2020). PD-1 blockade inhibits osteoclast formation and murine bone cancer pain. J. Clin. Investig..

[104] Wang Z., Jiang C., He Q., Matsuda M., Han Q., Wang K., Bang S., Ding H., Ko M.C., Ji R.R. (2020). Anti-PD-1 treatment impairs opioid antinociception in rodents and nonhuman primates. Sci. Transl. Med..

[105] Martins F., Sofiya L., Sykiotis G.P., Lamine F., Maillard M., Fraga M., Shabafrouz K., Ribi C., Cairoli A., Guex-Crosier Y. (2019). Adverse effects of immune-checkpoint inhibitors: Epidemiology, management and surveillance. Nat. Rev. Clin. Oncol..

[106] Abdelhakim S., Klapholz J.D., Roy B., Weiss S.A., McGuone D., Corbin Z.A. (2022). Mononeuritis multiplex as a rare and severe neurological complication of immune checkpoint inhibitors: A case report. J. Med. Case Rep..

[107] Patel A.S., Snook R.J., Amikar S. (2019). Chronic inflammatory demyelinating polyradiculoneuropathy secondary to immune checkpoint inhibitors in melanoma patients. Discov. Med..

[108] Koldenhof J.J., van der Baan F.H., Verberne E.G., Kamphuis A.M., Verheijden R.J., Tonk E.H., van Lindert A.S., van der Stap J., Teunissen S.C., Witteveen P.O. (2022). Patient-reported outcomes during checkpoint inhibition: Insight into symptom burden in daily clinical practice. J. Pain Symptom Manag..

[109] Kwatra S.G., Ständer S., Kang H. (2018). PD-1 Blockade-Induced Pruritus Treated with a Mu-Opioid Receptor Antagonist. N. Engl. J. Med..

[110] Ikoma A., Steinhoff M.S., Yosipovitch G., Schmelz M. (2006). The neurobiology of itch. Nat. Rev. Neurosci..

[111] Pinho-Ribeiro F.A., Verri W.A., Chiu I.M. (2017). Nociceptor Sensory Neuron-Immune Interactions in Pain and Inflammation. Trends Immunol..

[112] Ji R.R., Xu Z.Z., Gao Y.J. (2014). Emerging targets in neuroinflammation-driven chronic pain. Nat. Rev. Drug Discov..

[113] Gonçalves Dos Santos G., Delay L., Yaksh T.L., Corr M. (2020). Neuraxial Cytokines in Pain States. Front. Immunol..

[114] Vanderwall A.G., Milligan E.D. (2019). Cytokines in Pain: Harnessing Endogenous Anti-Inflammatory Signaling for Improved Pain Management. Front. Immunol..

[115] Ji R.R., Xu Z.Z., Strichartz G., Serhan C.N. (2011). Emerging roles of resolvins in the resolution of inflammation and pain. Trends Neurosci..

[116] Pannell M., Labuz D., Celik M.Ö., Keye J., Batra A., Siegmund B., Machelska H. (2016). Adoptive transfer of M2 macrophages reduces neuropathic pain via opioid peptides. J. Neuroinflamm..

[117] Celik M.Ö., Labuz D., Keye J., Glauben R., Machelska H. (2020). IL-4 induces M2 macrophages to produce sustained analgesia via opioids. JCI Insight.

[118] Laumet G., Ma J., Robison A.J., Kumari S., Heijnen C.J., Kavelaars A. (2019). T Cells as an Emerging Target for Chronic Pain Therapy. Front. Mol. Neurosci..

[119] Zhou X., Qiao G., Ren J., Wang X., Wang S., Zhu S., Yuan Y., Morse M.A., Hobeika A., Lyerly H.K. (2020). Adoptive immunotherapy with autologous T-cell infusions reduces opioid requirements in advanced cancer patients. Pain.

[120] Clauw D. (2017). Hijacking the endogenous opioid system to treat pain: Who thought it would be so complicated?. Pain.

[121] Blendon R.J., Benson J.M. (2018). The Public and the Opioid-Abuse Epidemic. N. Engl. J. Med..

[122] Boland J.W., Pockley A.G. (2016). Clinically relevant concentrations of opioids for in vitro studies. J. Opioid Manag..

[123] Rivat C., Ballantyne J. (2016). The dark side of opioids in pain management: Basic science explains clinical observation. Pain Rep..

[124] Santoni A., Arcuri E. (2020). The ambiguity of opioids revealed by immunology is changing the knowledge and the therapeutic approach in cancer and non-cancer pain: A narrative review. Immunol. Lett..

[125] Arcuri E., Mercadante S., Santoni A. (2021). Immunity and pain: Is it time for the birth of Immunoalgology?. Minerva Anestesiol..

